# Therapeutic Potency of Ovothiol A on Ethanol-Induced Gastric Ulcers in Wistar Rats

**DOI:** 10.3390/md21010025

**Published:** 2022-12-28

**Authors:** Amira Tarek Salaheldin, Mohamed Refaat Shehata, Hader I. Sakr, Tarek Atia, Ayman Saber Mohamed

**Affiliations:** 1 Department of Biotechnology, Faculty of Sciences, Cairo University, Giza 12613, Egypt; 2 Chemistry Department, Faculty of Sciences, Cairo University, Giza 12613, Egypt; 3 Department of Medical Physiology, Faculty of Medicine, Cairo University, Giza 12613, Egypt; 4 Department of Medical Physiology, Medicine Program, Batterjee Medical College, Jeddah 21442, Saudi Arabia; 5 Department of Medical Laboratory Sciences, College of Applied Medical Sciences, Prince Sattam bin Abdulaziz University, Al-Kharj 16273, Saudi Arabia; 6 Zoology Department, Faculty of Science, Cairo University, Giza 12613, Egypt

**Keywords:** ovothiol A, sea urchin, natural product, gastric ulcer, oxidative stress, inflammation

## Abstract

Peptic ulcer is a widespread disease, with a lifetime frequency of 5–10% among the general population and an annual incidence of 0.1–0.3%. Ovothiol A is naturally produced from sea urchin eggs with special antioxidant activity. Gastric ulcers were induced in rats by a single ethanol dose (5 mL/kg). The rats were divided into control, ulcer, and ulcer with 250 and 500 mg/kg ovothiol A doses. Molecular docking studies were used to examine the interactions between ovothiol A and the H+/K+ ATPase active site residues. Ovothiol A led to a significant decline (*p* < 0.05) in gastric juice volume, ulcer index, MDA, IL-6, and cytochrome c, while levels of gastric juice pH, GSH, CAT, GST, SOD, and NO increased. Histopathological investigation of stomach sections revealed architecture preservation of the gastric mucosa after ovothiol A administration. The anti-ulcerogenic activity of ovothiol A includes scavenging free radicals, inhibition of inflammation, regulation of apoptosis, and stabilization of fibroblast growth factors to promote gastric ulcers healing.

## 1. Introduction

Peptic ulcer is a lesion of the digestive system caused by gastric acid that typically develops in the stomach or proximal duodenum [1]. Peptic ulcer disease (PUD) is widespread, with a lifetime frequency of 5–10% among the general population and an annual incidence of 0.1–0.3% [2]. In 2019, there were nearly 8 million prevalent incidences of PUD [3].

PUD is due to a mismatch between defensive factors (mucus secretion, non-enzymatic and enzymatic antioxidants, surface active phospholipids, blood flow, prostaglandins, and cell renewal) and offensive agents such as (gastric acid, pepsin and reactive oxygen species (ROS) [4]. However, the two most important factors that cause an imbalance between the acid and the mucus are *H. pylori* infection, non-steroidal anti-inflammatory medicines (NSAIDs), and a rise in alcohol and smoking misuse [5,6,7,8,9].

The commonly used proton pump inhibitors (PPIs) and histamine receptor type-2 antagonists have shown side effects and several medication interactions [10]. Therefore, the need to face these challenges has encouraged research reports to explore new active compounds from natural sources [11].

The incredible biodiversity of sea environment creatures contributed to the identification of numerous new chemicals, the biological features, and technological applications of which are being thoroughly researched [12,13]. More attention has recently been paid to marine-isolated ovothiols (1-N-methyl-4-mercaptohistidine) from invertebrates, algae, and protozoa [14]. The three types of ovothiols (A, B, and C) differ in the nitrogen methylation of the aminoacidic side chain (non-, mono- and di-methylated, respectively).

Ovothiol A was first found in some echinoderm’s eggs (for example, the sea urchin *Paracentrotus lividus*) and in some mollusks and polychaetes biological fluids [15]. The thiol group of all ovothiols on their histidine imidazole ring has exceptional antioxidant characteristics [16,17,18]. Only the anionic thiolate form of thiols functions well as an electron donor, and ovothiol A is almost totally present in this form, giving it its special natural antioxidant capabilities. An in-vitro endothelial dysfunction model revealed hyperglycemic-induced anti-inflammatory and antioxidant effects of ovothiol A’s disulphide form [15,19].

The present study aims to investigate the therapeutic potential of ovothiol isolated from sea urchin eggs against an ethanol-induced peptic ulcer in rats.

## 2. Results

### 2.1. Molecular DFT Calculation of Ovothiol A

**Figure 1:**
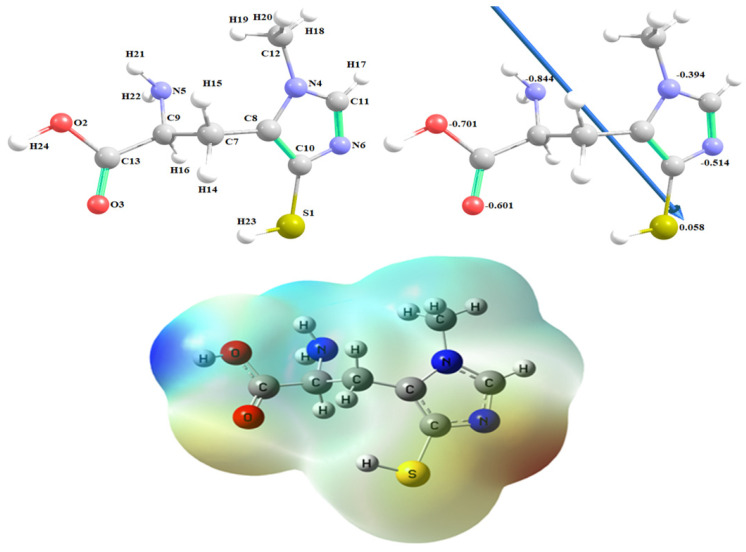
The optimized structure of ovothiol A, the vector of the dipole moment, the natural charges on atoms and molecular electrostatic potential (MEP) surface by density function B3LYP/6-311G++ (dp).

Figure 1 demonstrates ovothiol A’s optimized structures as the lowest energy configurations. The dipole moment and the natural charges obtained from Natural Bond Orbital Analysis (NBO) are shown on the active sites of oxygen, nitrogen, and sulfur atoms. The MEP loosely or excess electrons) charged electrostatic potential in the molecule. Both bond lengths and angles are shown in Table 1.

**Figure 2:**
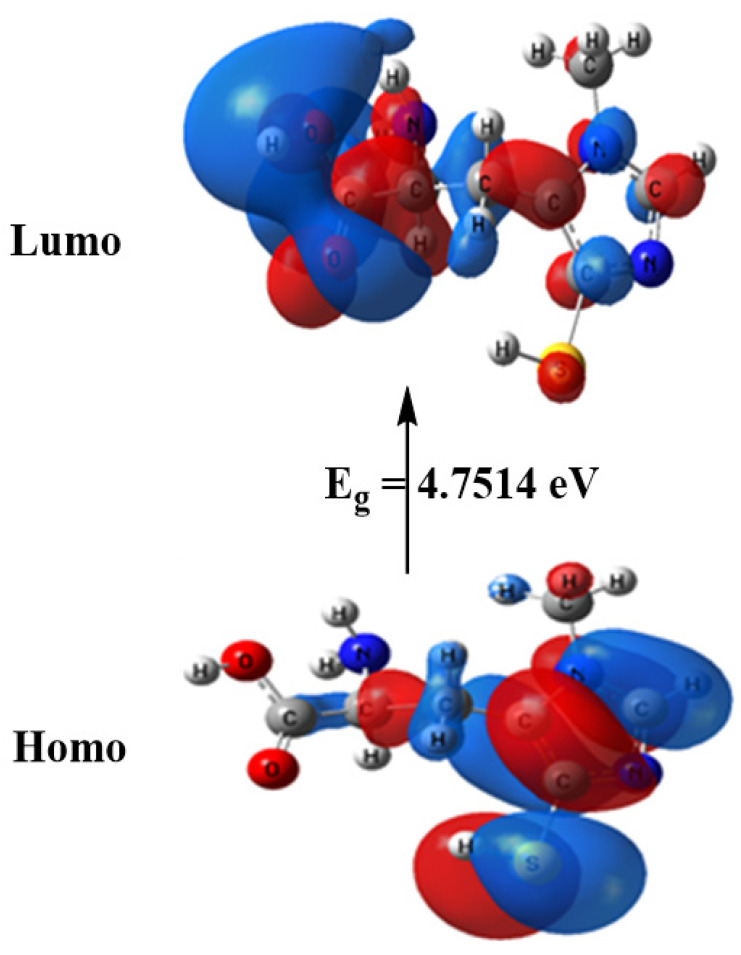
HOMO and LUMO charge density maps of B3LYP/6-311G++ (dp).

The computed total energy of ovothiol A is −986.465 Hartree, the highest occupied molecular orbital (HOMO) energy is −5.6010 eV, the lowest unoccupied molecular orbital (LUMO) energy is −0.8496 eV and the dipole moment is 6.9314 Debye. The higher total energy negativity indicates compound stability. Also, the energy gap (Eg) = ELUMO − EHOMO = 4.7514 eV, Figure 2 and Table 2. Many reactivity descriptors such as ionization potential (I), electron affinity (A), Electronegativity (χ), chemical potential (μ), hardness (η), softness (S) and electrophilicity index (ω), all derived from the HOMO and LUMO energies, proposed to comprehend many reactivity-related features of chemical processes, Table 2.

### 2.2. Molecular Docking Interaction between Ovothiol A with 1AFC (PDB ID: 1AFC)

**Figure 3:**
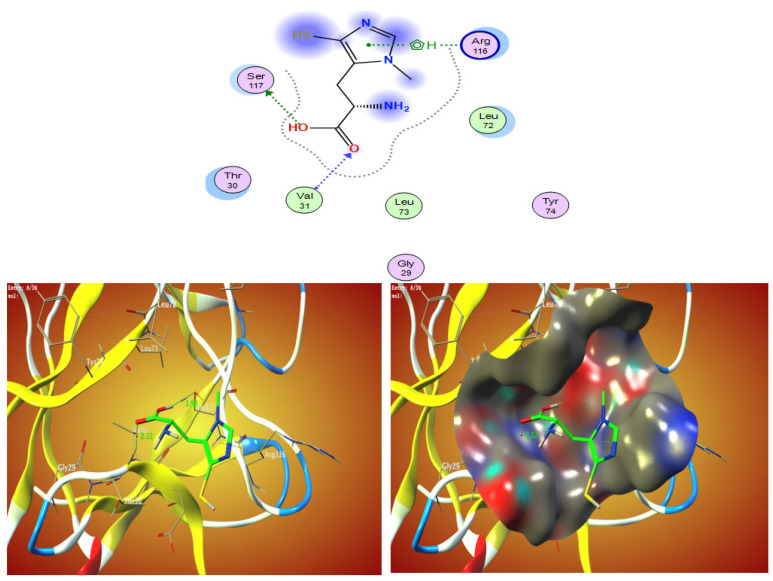
The 2D and 3D molecular docking simulation studies of the interaction between ovothiol A with 1AFC. Hydrophobic interactions with amino acid residues are shown with dotted curves.

The free energy binding of ovothiol A’s to the active sites of the 1AFC receptor was determined in the current investigation to be −4.3 kcal/mol, according to Table 3. The stronger the binding, the more negative the binding energy (Table 3 and Figure 3).

### 2.3. Ulcer l Markers

**Figure 4:**
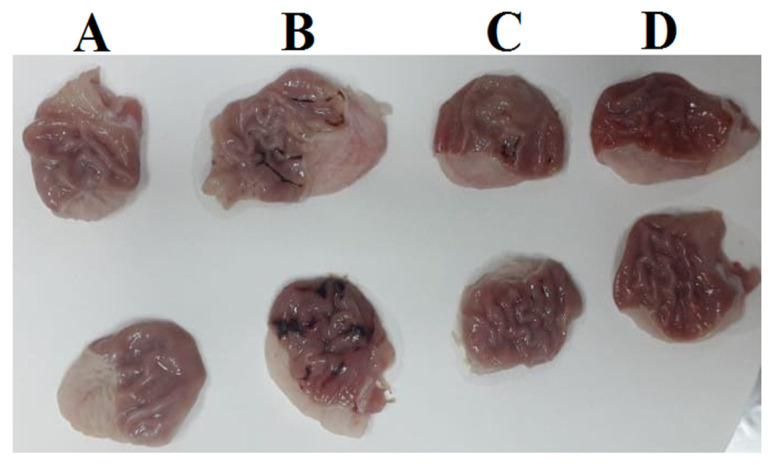
Stomachs of; control rat (**A**), ulcer rat (**B**), ovothiol A (250 mg/kg) rat (**C**) and ovothiol A (500 mg/kg) rat (**D**).

The gastric juice volume and ulcer index were significantly elevated (*p* < 0.05) while the pH was significantly reduced (*p* < 0.05) in the ulcer group compared to the control group. However, the ovothiol A administration with both concentrations caused a significant decrease (*p* < 0.05) in gastric juice volume and ulcer index compared to the ulcer group, as shown in Table 4 and Figure 4.

### 2.4. Markers of Oxidative Stress

Gastric MDA level was significantly increased (*p* < 0.05), while GSH and GST levels were significantly reduced (*p* < 0.05) in the ulcer group compared to the control group. Moreover, ovothiol A treatment significantly (*p* < 0.05) decreased MDA levels and elevated (*p* < 0.05) GSH and GST levels compared to the ulcer group, as shown in Table 5.

### 2.5. NO, PGE-2, IL6, and Cytochrome C

Table 6 showed significant ethanol-induced elevation (*p* < 0.05) in the cytochrome c, PGE-2 and IL6 levels compared to the control group. The levels of the aforementioned parameters were restored near the normal level by the administration of ovothiol A with its two doses.

### 2.6. Histopathological Analysis

**Figure 5:**
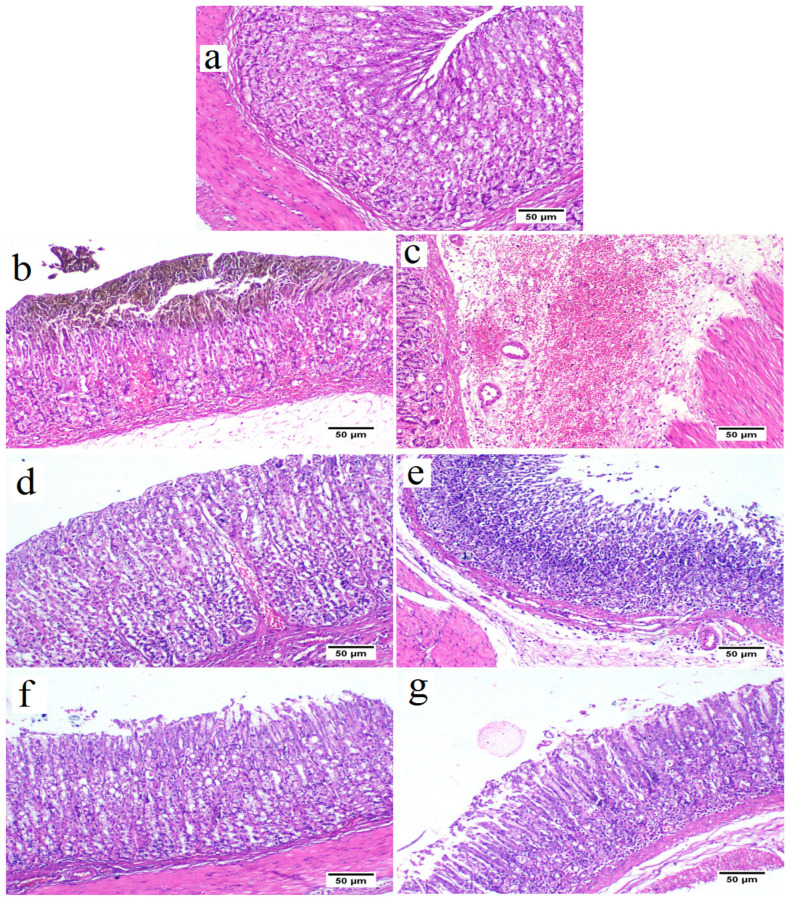
Stomach from control group (**a**) showing normal histological structure of stomach tissue that consisted of mucous epithelium covering gastric acini (H&E stain). Stomach from ulcer group showing destruction of the mucosal covering associated with extensive hemorrhage (**b**), and expansion of the submucosal layer with edema, and diffuse extensive hemorrhage (**c**). Stomach from ovothiol A (250 mg/kg) treated group showing apparently normal gastric mucosa (**d**) with mild to moderate submucosal edema and lowered number of mononuclear cells infiltration (**e**). stomach from ovothiol A (500 mg/kg) treated group showing apparently normal gastric mucosa (**f**) and few mononuclear cells infiltration with mild submucosal edema (**g**).

The control group revealed a standard histological structure characterized by a long tubular gastric gland (acini) in the mucosal layer that extended deep into the lamina propria and was covered by simple columnar epithelium (Figure 5a). The gastric acini showed the digestive enzyme-secreting zymogenic cells (chief cells) and the gastric acid-secreting parietal cells (oxyntic cells). The submucosal layer showed the blood vessels, nerves and ganglionated plexus within loose connective tissue. The muscular layer was well developed, with serosal covering the outer surface of the stomach (visceral peritoneum).

In contrast, the ethanol in the ulcer group triggered a severe gastric injury (Figure 5b,c). The mucosal layer showed severe alteration and loss of the epithelial cells with hemorrhages and congested blood capillaries. The lamina epithelialis and the lamina propria were infiltrated with inflammatory cells. Some examined areas presented severe necrosis of the gastric acini with mononuclear cell infiltration. The submucosal layer was severely expanded with the dispersion of the connective tissue by inflammatory edema and severely dilated blood vessels associated with extensive hemorrhage.

The low dose (250 mg/kg) of ovothiol A reduced the histopathologic scores, attenuating gastric damage, and inflammatory cell infiltration (Figure 5d,e) and decrease gastric hemorrhage with architecture preservation of the gastric mucosa in other examined sections.

**Figure 6:**
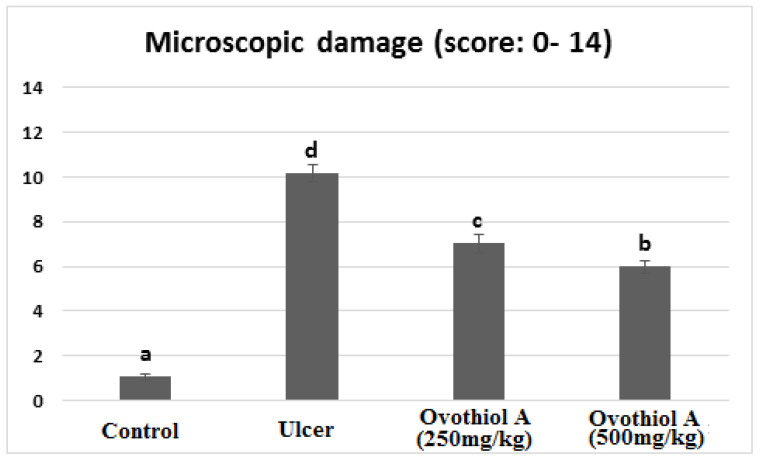
Histopathological score of stomachs of control, ulcer, ovothiol A (250 mg/kg) and (500 mg/kg). Data are expressed as the mean ± SE. a, b, c and d above the error bar indicate a significant difference.

Rats received a high dose of (500 mg/kg) ovothiol resulted in the highest protection of the gastric histological structure (Figure 5f,g). Few examined sections showed a reduction of mononuclear inflammatory cells compared with the ulcer group and low dose ovothiol treated group. Most examined sections revealed normal glandular gastric mucosal surface. The total histopathological lesion score showed the peak value recorded by the ulcer group that a significant difference from other experimental groups (Figure 6). The high dose of ovothiol showed a better improvement and scored fewer histopathological lesions compared with a low dose of the same drug.

## 3. Discussion

Major complications such as hemorrhage, perforation, or stomach outlet obstruction affects roughly 25% of people with peptic ulcer disease [3]. People worldwide are still suffering from peptic ulcer complications and looking for safe and effective treatment. The immense potential of marine natural products has been recently revealed by developments in the discovery, approval, and therapeutic use of marine pharmaceuticals. This work investigated the anti-inflammatory and antioxidant potential of ovothiol A extracted from sea urchins against ethanol-induced gastric ulcers.

In the current study, ethanol administration increased gastric juice secretion and ulcer index and decreased pH in rats. Acid secretion, oedema, haemorrhage, necrosis, and inflammation all contributed to higher gastric juice volume and gastric ulcer index in the ulcer group, as confirmed by the histopathological investigation. The mucous membrane of the digestive tract easily absorbs ethanol. High ethanol concentrations would damage the gastric mucosa and may cause hemorrhagic gastritis within 30 min [20]. Ethanol and its byproduct, acetaldehyde, can damage the gastric mucosa’s epithelial and vascular endothelial cells [21,22,23]. Ovothiol A (250 and 500 mg/kg) showed a therapeutic effect against gastric ulcers indicated by a significantly elevated gastric pH with decreased gastric juice volume and gastric ulcer index.

Under typical physiological conditions, the body has a built-in system to maintain a healthy balance between oxidation and antioxidation [24]. One of the main reasons why gastric ulcers happen is because this system is out of balance [25]. Malondialdehyde (MDA) is the major diagnostic for evaluating lipid peroxidation. In line with prior findings, the administration of ethanol in rats resulted in a considerable elevation in gastric issue’s MDA levels [26,27]. High ethanol intake produces substantial ROS, which react with membrane lipids to produce lipid peroxides [28]. Several investigations have indicated that antioxidant substances that scavenge free radicals prevent ethanol-induced stomach ulcers [26,29]. In our study, the rat treated with ovothiol A (250 and 500 mg/kg) showed a decrease in MDA levels which indicated the antioxidant activity of ovothiol.

According to our results, ulcer groups showed a significant decrease in rats’ antioxidant system (GSH, CAT, GST and SOD), which is considered the initial defense against ethanol-induced free radical damage to gastric mucosal cells [30]. Because of the antioxidant activity of ovothiol A [31] the gastric tissues restored the antioxidant system levels.

Prostaglandin E2 (PGE2) and nitric oxide (NO) are essential mediators for maintaining gastric mucosal defense integrity and healing gastric ulcers [32]. In the current study, ethanol caused a significant decrease in NO and PGE2 levels. NO is a vasodilator factor that keeps the integrity of the gastric epithelial and mucus barriers by regulating blood flow [33]. Most importantly, NO is an important part of angiogenesis and tissue regeneration and could help heal ulcers [34]. Also, several studies found that NO plays a protective role in peptic ulcers and can speed gastric ulcer healing [35,36]. PGE2 is one of the endogenous gastric mucosa protective factors by increasing the blood flow, enhancing mucus and bicarbonate secretion, and making epithelial cells more resistant to stimuli [37]. Having oxidative damage present, which causes prostaglandins to be converted into oxidation products like 8-isoprostaglandin F2alpha, was thought to be responsible for ethanol’s suppressive effect on gastric mucosal PGE2 level [38,39].

However, treatment with ovothiol A (250 and 500 mg/kg) increased NO and PGE2 levels that protect the stomach lining against ethanol-caused ulcers.

Interleukins are important regulators of the mucosal defense barrier [40]. There is evidence that ethanol may stimulate the innate immune system, changing the levels of inflammatory cytokines, including Interleukin 6 (IL-6) [41,42]. The current data demonstrated ethanol-induced elevated levels of IL-6 in the gastric tissue that could activate oxidative stress, drive neutrophils, lymphocytes, and phagocytes in the inflammatory areas and create toxic metabolites and lysosomal enzymes, injuring the gastric mucosa [43]. Our findings showed that ovothiol A treatment with different doses decreased the IL-6 level

Moreover, apoptosis is a key mechanism in multicellular organisms for maintaining homeostasis and responding to environmental stimuli. The release of many substances from mitochondria mediates apoptosis. The interaction of the pro-apoptotic protein’s caspase-3 and -9 regulates the release of cytochrome c that may turn on or off apoptosis [44]. In the current study, ethanol administration increased cytochrome C levels in rats. It was reported that ethanol stimulated the mitochondrial signaling pathway of apoptosis in the gastric mucosa [45]. The release of cytochrome c was linked to ROS production such as H_2_O_2_ [46,47]. In our study, ovothiol A treatment (250 and 500 mg/kg) decreased the cytochrome C levels, indicating its effective gastroprotective activity. Furthermore, Zhu et al. [48] reported that fibroblast growth factors (FGFs) binding to their receptors more effectively if they are bound to sulfate. According to the current study, ovothiol A promotes the healing of gastric ulcers by stabilizing FGFs to prevent denaturation in the stomach’s acidic pH.

## 4. Materials and Methods

### 4.1. Drugs and Chemicals

All solvents, chemicals and drugs were purchased from Sigma Aldrich Co. (St. Louis, MO, USA).

### 4.2. Isolation of Ovothiol A from Sea Urchin Eggs

Ovothiol-A was isolated using the Russo et al. technique [49]. Prior to setting the sea urchin on the water’s surface, we injected its eggs with about 1 mL of KCl. The gathered sea urchin eggs were centrifuged at 2000 RPM for 10 min, and the supernatant was discarded. The eggs were crushed and agitated for 12 h in a mixture of 20 mL 1 M HCl and 80 mL ethanol. At 4 °C the homogenate was centrifuged for 15 min at 6000 RPM. A rotatory evaporator was used to evaporate the ethanol (at 40 °C), and the supernatant was then gathered. The lipids were eliminated from the mixture using 50 mL of diethyl ether. Through the use of an alumina column, peroxide is eliminated from the solution. The Dowex 50WX2 (1 cm × 22 cm) column was loaded with the acidic solution. Water, 0.1 M, 0.5 M, and 4 M HCl were used as elusions. The 4M fraction’s ovathiol was converted to ovathiol disulfide after being exposed to air for 4 h at a pH of 8. Before repeating the chromatography on the same Dowex column, the pH of the solution was brought down to 2. A 40 °C oven was used to dry the crystals, which resulted in colorless, glassy solid crystals. 3.5 mg of ovathiol-A is produced from each 10 g eggs. For characterization details of ovathiol-A, please refer to the Supplementary Materials.

### 4.3. Ovothiol-A Molecular DFT Calculation

Using the Gaussian 09 programme, density functional theory (DFT) simulations were performed to examine the equilibrium geometry of Ovathiol A at the B3LYP/6-311G++ (dp) level of theory.

### 4.4. Molecular Docking Interaction between Ovothiol A with 1AFC (PDB ID: 1AFC)

Utilizing the MOA2019 programme, molecular docking studies were conducted to determine the potential binding modes for the receptor 1AFC’s most active location. The proton-pump inhibitor drugs target 1AFC, which is gastric H+,K+ ATPase (the gastric acid pump).

### 4.5. Experimental Animals

Twenty-four male Wister rats Rattus Norvegicus with similar age (± one week) and weight (130–150 (±2 g) were used in the experiments. Steel-wire topped polycarbonate boxes were used for animal housing and were bedded with wood shavings. The animals were provided with a standard laboratory diet, and water ad libitum and kept under fixed housing and handling conditions.

### 4.6. Ethical Consideration

The experimental techniques and practices of the study were approved by the Faculty of Science Institutional Animal Care and Use Committee (IACUC) at Cairo University, Egypt. Under ethical approval number CUIF3120, all of the experiments were done in line with international rules for the care and use of laboratory animals.

### 4.7. Induction of Peptic Ulcer

All animals fasted 24 h before administration of ethanol (5 mL/kg of body weight, orally) [50].

### 4.8. Experimental Design

The rats were divided into four equal groups (*n* = 6):

Group I (control group): Rats were administrated orally with dist. water (5 mL/kg), then after one hour, dist. water was administrated again.
Group II (ulcer group): Rats were administrated orally with ethanol (5 mL/kg), then after one hour, dist. water was administrated again.
Group III (ovothiol A-500): Rats were administrated orally with ethanol (5 mL/kg), then after one hour, ovothiol A (500 mg/kg) [31] was administrated.
Group IV (ovothiol A-250): Rats were administrated with ethanol 5 mL/kg orally, then after one hour, ovothiol A (250 mg/kg) was administrated.

### 4.9. Animal Handling and Specimen Collection

Animals were euthanized using 3% sodium pentobarbital (100 mg/kg) overdose one hour after the last treatment [51]. The stomach was then collected and blotted with filter paper. For the biochemical study, a section of the stomach was frozen at −80 degrees Celsius. Another section of the stomach was fixed before histological analysis by suspending it in 10% formal saline.

### 4.10. Ulcer Markers

After collecting the gastric juice by cutting open the stomachs, the samples were centrifuged for 10 min at 3000 rpm to remove the solids from the liquid, and the supernatant volume was measured. A pH meter was used to measure the pH level of the gastric juice. Stomachs were inspected for ulcers using magnification (×10). Methods used to evaluate the ulcer index included: Below 1 mm = 1 point, 1–2 mm = 2 points, and >3 mm = 3 points for ulcer length. The ulcer index was calculated by taking the total number of points and dividing it by 10 (lens magnification) [52].

### 4.11. Stomach Homogenate Preparation

10% *w*/*v* of stomach tissue was homogenized in ice-cold 0.1 M Tris-HCl buffers (pH 7.4). The homogenate was centrifuged at 3000 rpm at 4 °C for 15 min, and the supernatant was biochemically analyzed.

### 4.12. Biochemical Parameters

Malondialdehyde (MDA), superoxide dismutase (SOD), catalase (CAT), nitric oxide (NO) reduced glutathione (GSH), and glutathione-S-transferase (GST) were determined following the manufacturer’s instructions. Interleukin 6 (IL-6), prostaglandin E2 (PGE2) and cytochrome c were determined using the relevant enzyme-linked immunosorbent assay (ELISA) kit following the manufacturer’s instructions.

### 4.13. Histopathological Analysis

For each rat, a small gastric tissue section was fixed with 10% neutral buffered formalin, embedded in paraffin, cut into 5 μm thickness and stained with hematoxylin and eosin stain. The specimens were examined under Olympus BX43 light microscope, and sections were captured by Olympus DP27 camera connected to Cellsens dimensions software (Olympus). A 0–14 range was used to score the microscopic damage according to [53,54], where epithelial cell loss or the presence of inflammatory cells scored 0–3 and oedema in the upper mucosa or hemorrhagic damage scored 0–4. The total microscopic score was obtained by summating the four histopathological scores.

### 4.14. Statistical Significance

The statistical analysis was performed using SPSS for Windows (version 15.0). Data were expressed in the form of means ± standard error of the mean (SEM). The one-way analysis of variance (ANOVA) was used to perform within-group comparisons, and the Duncan post hoc test was used to compare the group means. *p* values < 0.05 were considered statistically significant.

## 5. Conclusions

The current study revealed that ovothiol A exhibited antiulcerogenic activity by reducing gastric juice volume, ulcer index, MDA, IL-6, and cytochrome c, increasing gastric juice pH, GSH, CAT, GST, SOD, and NO, and improving gastric mucosa architecture. The therapeutic pathways of ovothiol A include scavenging free radicals, inhibition of inflammation, regulation of apoptosis, and promotion of the healing of stomach ulcers by stabilizing fibroblast growth factors.

## Figures and Tables

**Table 1:** Selected optimized bond lengths (Å) and bond angles (°) of ovothiol A.

**Type of Bond**	**Bond Length (Å)**	**Type of Bond**	**Bond Length (Å)**
S1-C10	1.777	N6=C11	1.316
S1-H23	1.348	C7-C8	1.492
O2-C13	1.357	C7-C9	1.560
O2-H24	0.970	C7-H14	1.094
O3=C13	1.205	C7-H15	1.095
N4-C8	1.391	C8=C10	1.342
N4-C11	1.360	C9-C13	1.530
N4-C12	1.455	C9-H16	1.092
N5-C9	1.459	C11-H17	1.080
N5-H21	1.015	C12-H18	1.090
N5-H22	1.014	C12-H19	1.090
N6-C10	1.367	C12-H20	1.093
**Type of Angle**	**Angle (°)**	**Type of Angle**	**Angle (°)**
C10-1-23	96.80	N5-C9-C13	116.4
S1-C10-N6	118.1	N5-C9-H16	108.6
S1-C10-C8	130.5	H21-N5-H22	107.0
C13-O2-24	107.3	C10-N6-C11	105.0
O2-C13-O3	122.5	N6-C10-C8	111.3
O2-C13-C9	113.1	N6-C11-H17	125.5
O3-C13-C9	124.4	C8-C7-C9	113.6
C8-N4-C11	106.9	C8-C7-H14	108.7
C8-N4-C12	127.1	C8-C7-H15	111.1
N4-C8-C7	125.0	C7-C8-C10	130.7
N4-C8-C10	104.3	C9-C7-H14	108.4
C11-N4-12	126.0	C9-C7-H15	108.4
N4-C11-N6	112.5	C7-C9-C13	108.0
N4-C11-H17	122.0	C7-C9-H16	107.2
N4-C12-H18	108.4	H14-C7-H15	106.3
N4-C12-H19	109.9	C13-C9-H16	105.4
N4-C12-H20	110.9	H18-C12-H19	109.5
C9-N5-H21	110.7	H18-C12-H20	108.4
C9-N5-H22	111.1	H19-C12-H20	109.7
N5-C9-C7	110.8		

**Table 2:** Calculated energies and the properties of ovothiol A.

Property	Ovothiol A	Equation	Ovothiol A
The total energy E (a.u.)	−986.465	Eg = ELUMO−EHOMO (eV)	4.7514
EHOMO (eV)	−5.6010	Ionization potential: I = −EHOMO	5.6010
ELUMO (eV)	−0.8496	Electron affinity: A = −ELUMO	0.8496
Dipole moment (Debye)	6.9314	chemical softness: S = 1/2η	0.2104
Electronegativity: χ = (I + A)/2	3.2253	chemical potential: μ = −χ	−3.2253
Chemical hardness: η = (I − A)/2	2.3757	Electrophilicity: ω = μ^2^/2η	2.1894

**Table 3:** The Docking interaction data calculations of ovothiol A with 1AFC (PDB ID: 1AFC).

	Receptor			Interaction	Distance (Å) *	E (kcal/mol)
O2	OG	SER	117	H-donor	2.91 (1.98)	−2.6
O3	N	VAL	31	H-acceptor	3.08 (2.12)	−1.2
5-ring	CG	ARG	116	pi-H	3.54	−0.5

* The lengths of H-bonds are in brackets.

**Table 4:** The curative potency of ovothiol A on the gastric ulcer markers in rats.

Groups	pH	Volume (mL)	Ulcer Index
Control	5.80 ± 0.12 ^d^	1.50 ± 0.37 ^a^	-
Ulcer	2.80 ± 0.21 ^a^	4.83 ± 0.26 ^c^	0.91 ± 0.05 ^c^
Ovothiol A (250 mg/kg)	3.77 ± 0.13 ^b^	3.08 ± 0.36 ^b^	0.49 ± 0.03 ^b^
Ovothiol A (500 mg/kg)	5.52 ± 0.18 ^c^	2.96 ± 0.17 ^b^	0.33 ± 0.04 ^a^

Values are mean ± SEM (*n* = 6). Values with different superscript letters ^a–d^ are significantly different (*p* < 0.05).

**Table 5:** The curative potency of ovothiol on oxidative stress markers of ethanol-induced gastric ulcer induced in rats.

Groups	MDA (nmol/g Tissue)	GSH (mg/g Tissue)	GST (U/g Tissue)	SOD (U/g Tissue)
Control	1.27 ± 0.03 ^a^	1.32 ± 0.05 ^d^	0.53 ± 0.03 ^c^	29.60 ± 1.13 ^c^
Ulcer	2.31 ± 0.07 ^d^	0.29 ± 0.01 ^a^	0.11 ± 0.02 ^a^	16.74 ± 1.03 ^a^
Ovothiol A (250 mg/kg)	1.84 ±0.05 ^c^	0.49 ± 0.03 ^b^	0.33 ± 0.04 ^b^	21.34 ± 1.04 ^b^
Ovothiol A (500 mg/kg)	1.43 ± 0.03 ^b^	0.63 ± 0.04 ^c^	0.41 ± 0.05 ^b^	27.13 ± 1.64 ^c^

Values are mean ± SEM (*n* = 6). Values with different superscript letters ^a–d^ are significantly different (*p* < 0.05).

**Table 6:** The curative potency of ovothiol on Apoptosis and inflammatory parameters in Ethanol-induced gastric ulcer in rats.

Groups	Cytochrome C (ng/mg Tissue)	IL-6 (pg/mg Tissue)	PGE_2_ (pg/mg Tissue)	NO (µmol/g Tissue)
Control	1.65 ± 0.04 ^a^	1.81 ± 0.08 ^a^	3.17 ± 0.58 ^c^	460.05 ± 15.78 ^c^
Ulcer	2.77 ± 0.26 ^c^	2.67 ± 0.28 ^c^	1.97 ± 0.32 ^a^	347.23 ± 11.91 ^a^
Ovothiol A (250 mg/kg)	2.26 ± 0.08 ^b^	2.24 ± 0.11 ^b^	2.42 ± 0.10 ^b^	405.93 ± 13.17 ^b^
Ovothiol A (500 mg/kg)	2.06 ± 0.06 ^b^	1.92 ± 0.08 ^a^	2.92 ± 0.24 ^c^	442.82 ± 19.92 ^c^

Values are mean ± SEM (*n* = 6). Values with different superscript letters are significantly different (*p* < 0.05).

## Data Availability

Not applicable.

## Supplementary Materials

The following supporting information can be downloaded at: https://www.mdpi.com/article/10.3390/md21010025/s1, Supplementary Material: Characterization of ovothiol A.

## Abbreviations

PUD	Peptic ulcer disease
ROS	Reactive oxygen species
NSAIDs	Non-steroidal anti-inflammatory medicines
PPIs	Proton pump inhibitors
MEP	Molecular electrostatic potential
MDA	Malondialdehyde
PGE2	Prostaglandin E2
NO	Nitric oxide
SOD	Superoxide dismutase
CAT	Catalase
GSH	Reduced glutathione
GST	Glutathione-S-transferase
IL-6	Interleukin 6
FGFs	Fibroblast growth factors
*H. pylori*	*Helicobacter pylori*
