# Supplementary material for: Therapeutic Potency of Ovothiol A on Ethanol-Induced Gastric Ulcers in Wistar Rats
Source: Mar Drugs. 2022 Dec 28;21(1):25. doi: 10.3390/md21010025 (PMC9862145; doi:10.3390/md21010025)
Supplement: Supplementary file 1 [file marinedrugs-21-00025-s001.zip › marinedrugs-2094170-supplementary.pdf]

Characterization of Ovothiol A

1. Material and methods

1.1. InfraRed spectroscopy (JASCO FT 761 photometer at SIC-SFRC)

The functional groups of Ovothiol A were determined using infrared spectroscopy. The sample was mixed with KBr disc. The IR spectra were executed at a resolution of 1 cm^{-1} (from 4000 to 400 cm^{-1}).

1.2. Mass Spectroscopy

Shimadzu QP 2010 Plus (Kyoto, Japan) was used for LC-MS analysis. The electron energy was 70 eV. A mass range from 50 to 500 m/z was scanned at a rate of 1000 amu/s, which was corresponded to 0.5 event time. The ion source temperature was 250 °C.

1.3. High-performance liquid chromatography (HPLC) Analysis

An Agilent (USA) 1200 Series system with a binary pump (G1312A), a thermostatic column compartment (G1316A), and a diodearray detector was used to conduct the HPLC analysis (DAD; G4212B). With a Zorbax Eclipse Plus C18 column (250 mm 4.6 mm, 5 m, Agilent) and ultra-pure water mobile phase as the eluent, chromatographic separation was performed at a flow rate of 1.0 mL/min at 30 °C. At 215 nm.

2. Results

2.1. IR spectra

The IR spectra of Ovothiol A (Figure S1) showed the peak at 3459.67 cm^{-1} is assigned to O–H stretching vibration. The peak 3231.32 cm^{-1} with strong intensity represents N–H stretching mode. The peak at 2364.30 cm^{-1} corresponds to the NH_2 group. The peak at 1637.27 cm^{-1} indicating the C=O stretching mode of vibration. The NH_2 bending vibrations occur at 1637.27and 873.59 cm^{-1}. The peaks at 1458.89 cm^{-1} correspond to the C=S stretching. The peak at 1127.19 cm^{-1} gives rise to C–N stretching mode of vibration. The spectra show absorption band in the region of 1046.19 cm^{-1} which are due to in-plane C–H bending vibration. C–C– N stretching vibration obtained at 873.59 cm^{-1}.

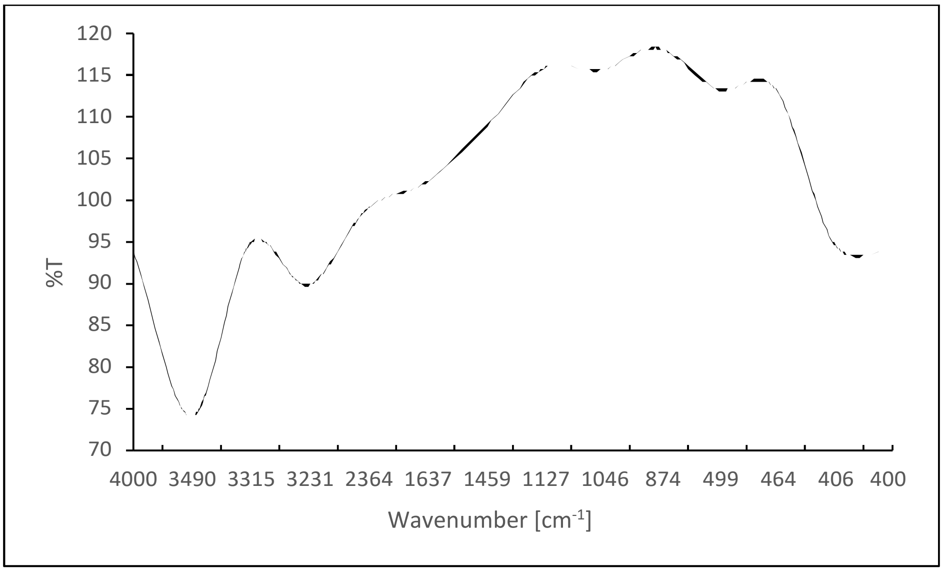

Figure S1. Infrared spectrum of ovothiol A.

2.2. The mass spectra

LC–MS of Ovothiol A showed remarkable peaks at (m/z) 401 corresponding to molecular weight of Ovothiol A (Figure S2).

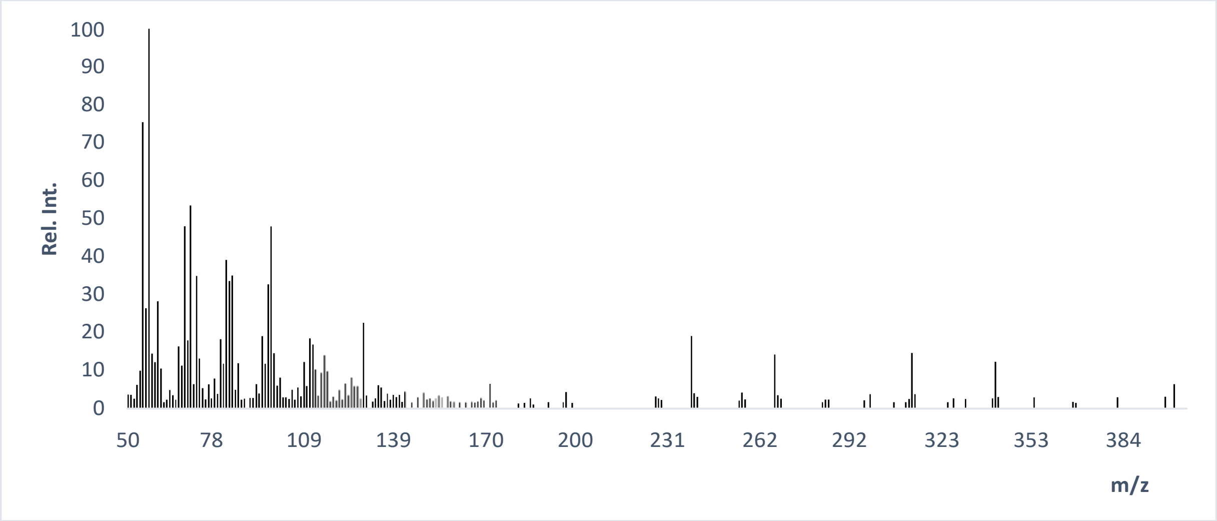

Figure S2. The mass spectra of ovothiol A.

2.3. HPLC

HPLC of the purified Ovothiol A showing the retention time at 4.87 min (Figure S3).

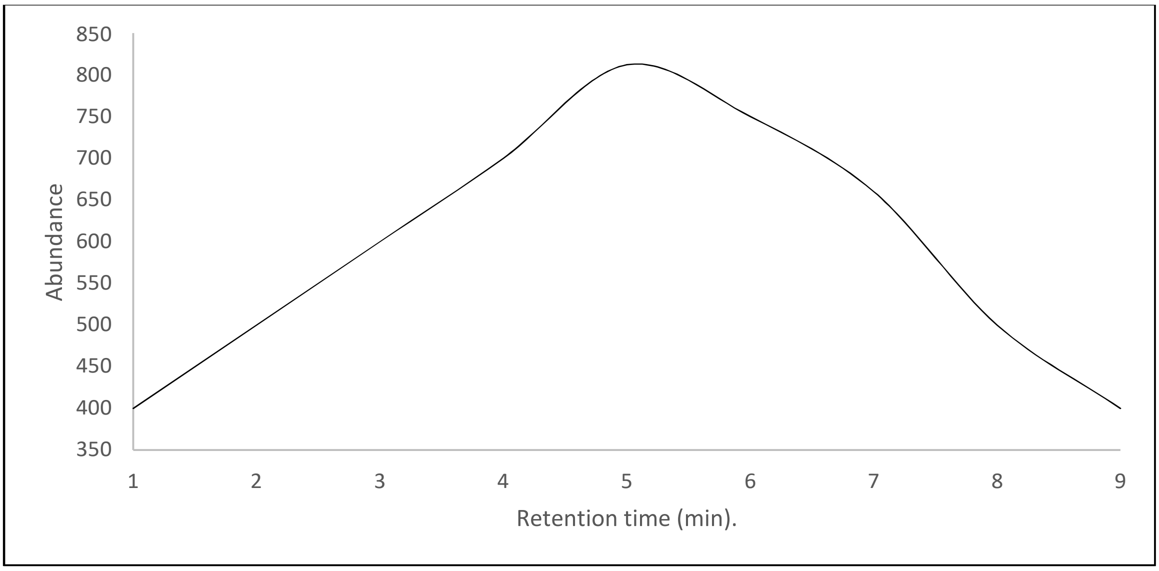

Figure S3. Thin layer chromatography (TLC) of the purified Ovothiol.

2.4. Physical properties of ovothiol A

Ovothiol A is a white crystal that is soluble in water and mineral acids (Table S1).

Table S1: The physical properties of Ovothio A.

Solubility	Soluble in water and Hydrochloric acid.<br>In soluble in methanol, ethanol, acetone, and dimethyl sulfoxide.
Appearance	Crystals
Color	White
